# Gut Dysbiosis and Muscle Aging: Searching for Novel Targets against Sarcopenia

**DOI:** 10.1155/2018/7026198

**Published:** 2018-01-30

**Authors:** Anna Picca, Francesca Fanelli, Riccardo Calvani, Giuseppina Mulè, Vito Pesce, Alex Sisto, Cecilia Pantanelli, Roberto Bernabei, Francesco Landi, Emanuele Marzetti

**Affiliations:** ^1^Department of Geriatrics, Neurosciences and Orthopedics, Catholic University of the Sacred Heart, Rome, Italy; ^2^Institute of Sciences of Food Production, National Research Council, Bari, Italy; ^3^Department of Biosciences, Biotechnology and Biopharmaceutics, University of Bari, Bari, Italy

## Abstract

Advanced age is characterized by several changes, one of which is the impairment of the homeostasis of intestinal microbiota. These alterations critically influence host health and have been associated with morbidity and mortality in older adults. “Inflammaging,” an age-related chronic inflammatory process, is a common trait of several conditions, including sarcopenia. Interestingly, imbalanced intestinal microbial community has been suggested to contribute to inflammaging. Changes in gut microbiota accompanying sarcopenia may be attenuated by supplementation with pre- and probiotics. Although muscle aging has been increasingly recognized as a biomarker of aging, the pathophysiology of sarcopenia is to date only partially appreciated. Due to its development in the context of the age-related inflammatory milieu, several studies favor the hypothesis of a tight connection between sarcopenia and inflammaging. However, conclusive evidence describing the signaling pathways involved has not yet been produced. Here, we review the current knowledge of the changes in intestinal microbiota that occur in advanced age with a special emphasis on findings supporting the idea of a modulation of muscle physiology through alterations in gut microbial composition and activity.

## 1. Introduction

Advances in medicine have led to worldwide population aging with an ever-growing proportion of elderly individuals. In such a scenario, strategies able to extend healthy lifespan and to foster active aging are a top public health priority. Indeed, advanced age is associated with an extraordinarily high prevalence of chronic disease conditions (e.g., cardiovascular disease, diabetes, cancer, and neurodegeneration), which in turn contribute to a number of negative health-related events (e.g., poor quality of life, morbidity, loss of independence, institutionalization, and mortality) [1].

The progressive loss of skeletal muscle mass and strength/function, referred to as sarcopenia, is increasingly recognized as a relevant determinant of negative health outcomes in late life [2]. As such, sarcopenia is endorsed as a meaningful biomarker allowing for the discrimination, at a clinical level, of biological from chronological age [3]. Despite growing interest surrounding the sarcopenia phenomenon, several limitations exist that impede its full appreciation in the clinical arena. Indeed, the lack of a univocal operational definition of sarcopenia and unbiased methods for assessing muscle mass and function represent major limitations in the field [3]. In addition to this, the incomplete knowledge of the pathophysiology of sarcopenia hampers the identification of targets that could be exploited for drug development [4].

A growing body of evidence suggests that the innumerable microorganisms that populate the mammalian gastrointestinal tract (gut microbiota) are tightly linked to the aging process of their host [5, 6]. Indeed, this microbial community, mostly composed of bacteria, participates in crucial activities of the gut barrier including the generation of metabolites essential for several host functions [7] and the mediation of exogenous chemical effects on their host [8].

Age-related changes in the bacterial composition of the microbiota are well known, and alterations of gut microbiota driven by the diet may affect the health of elderly people [9, 10]. However, the complexity of mammalian gut microbiota and the technical challenges in isolating specific “prolongevity” microbial variants limit the knowledge of the microbiota to taxonomic and metagenomic profiling. The functions of individual microbial genes and the molecular mechanisms through which they intervene in host aging are yet to be elucidated. Even less is known about the implications of microbiota-immune system crosstalk on muscle aging.

Here, we overview the current evidence supporting the involvement of gut microbiota in muscle aging. Special focus is placed on the analytical tools that may help capture the complexity of human microbiota and its crosstalk with several body systems in advanced age.

## 2. Microbiota in Health and Aging

The human gut microbiota is a complex ecosystem existing in a symbiotic and commensal relationship with 10–100 trillion microbial cells, mostly bacteria but also yeast, virus/phages, fungi, archaea, microeukaryotes, protozoa, helminths, and parasites [11].

Being frequently confused with the microbiota, the term microbiome indicates the gene catalogues these microbes harbor [12], sometimes referred to as our second genome [13].

The gut represents the largest contributor to the human microbiota. Although rich in variety throughout its segments, the human gastrointestinal tract harbors about 10^14^ bacterial cells, which is ten times the number of human cells in the body [14]. Such a bulk of biomass, with 3.3 million nonredundant genes, is almost 150 times the 23 thousand genes present in the cells of the human body and plays a central role in health [15, 16]. Despite a high degree of interindividual variability in gut microbiota composition [17, 18], there is a remarkable similarity in the basal gene metabolic activities across individuals [17].

The human gut controls luminal gastrointestinal content at the interface with the external environment and is involved in several host functions. The microbial ecosystem can impact nutrient absorption through bioconversion of food compounds and is also responsible for the nutritional status of the organism [19]. Indeed, among its activities, gut microbiota is involved in the production of micronutrients, such as essential vitamins and cofactors; regulation of the immune system; transformation of xenobiotics; breakdown of complex lipids, proteins, and polysaccharides into metabolite intermediates [e.g., short-chain fatty acids (SCFA)]; and waste product detoxification and finally represents a barrier against the spread of pathogens [20, 21].

In addition, the gut microbiota participates in host metabolism by contributing to bile acid metabolism and recirculation; absorption of calcium, magnesium, and iron; regulation of fat storage; and activation of bioactive compounds [22, 23].

The gut also serves as an endocrine, immune, and neuronal organ. As the largest endocrine organ, it releases hormones by means of enteroendocrine cells [24], but its role goes well beyond. Besides its barrier-like role that protects the host from pathogen colonization [25], the intestinal microbiota also participates in the development and homeostasis of the host immune system [26, 27]. Indeed, 70% of the body immune cells reside in the gut-associated lymphoid tissue. Immune cells can sense changes in the microbiota through specific gastrointestinal cells and receptors and, in turn, trigger lymphocyte accumulation and differentiation in the gastrointestinal tract [28]. The interaction between gastrointestinal cells and commensal bacteria fosters immunological tolerance or inflammatory responses to pathogens by regulating immune homeostasis in the gut [29]. This crosstalk between microbiota and gut mucosal cells (enterocytes, dendritic cells, lymphocytes, macrophages, and M cells) modulates the production of various cytokines and chemokines. These can be proinflammatory, such as interleukin (IL) 1 and 8, or anti-inflammatory, such as IL10 and transforming growth factor [30].

A bidirectional gut-brain communication involving the microbiota has also been recognized and comprises neural [e.g., enteric nervous system (ENS), vagus, and sympathetic and spinal nerves] and humoral pathways (e.g., cytokines, hormones, and neuropeptides as signaling molecules) [31]. Such a communication network is referred to as the “microbiome-gut-brain axis” [32] and signals gastrointestinal perception to the brain which in turn elaborates a gastrointestinal response.

Through this gut-brain homeostasis axis, the microbiota is able to influence numerous aspects of host health, including organ morphogenesis, immune system and gastrointestinal tract development and maturation, intestinal vascularization, tissue regeneration, carcinogenesis, metabolism [33], bone homeostasis [34], memory formation, emotional arousal, affective behavior [35], intuitive decision-making, and a range of neurological disease [32, 36].

The composition of the gut microbiota drastically changes during the first 2-3 years of life [37]. Primarily dominated by *Bifidobacteria* [38, 39], its development and intraindividual variation in healthy individuals is highly influenced by several factors including mode of delivery (vaginal or cesarean), diet, use of antibiotics, geography, and environmental exposure [40–42]. In adults (<65 years), the interindividual microbial diversity of the gut microbiota reaches its maximum, but a plateau effect is observed afterwards as a consequence of the aging process [37, 43, 44]. An adult-associated core microbiome comprising 66 dominant operational taxonomic units (OTUs) [45] that differs from the core and diversity levels of younger counterparts has been identified [30].

Among the age-associated changes in the microbial population, a reduced abundance of several butyrate producers (*Clostridium* clusters XIVa and IV) has been reported by both 16S targeted Sanger sequencing and next-generation pyrosequencing [46, 47].

**Figure 1:**
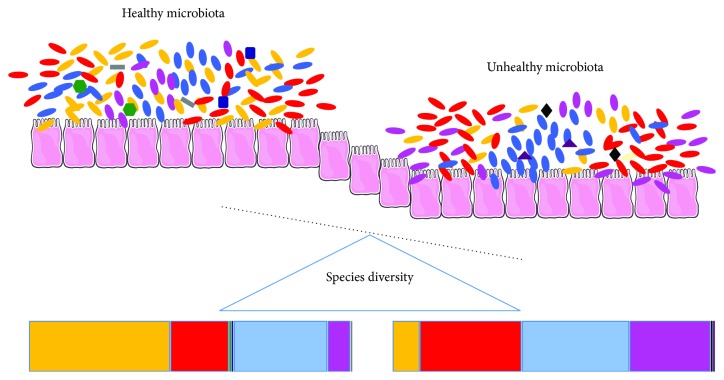
Healthy microbiota is a balanced community of symbiont, commensal, and pathobiont microorganisms. Each microbial class confers distinct characteristics to the host. Either imbalance in alpha-diversity or variations of relative abundance of single microbial taxa results in microbiota imbalance. As such, a sterile inflammation occurs and may predispose the host to opportunistic infections, ultimately leading to acute inflammation.

In addition, analysis of the microbial composition of 161 Irish people aged 65 years and older compared to nine younger controls showed that, even if possessing a unique individual microbiota profile, microbiota of older people was represented predominantly by Bacteroidetes population [48], as inferred by pyrosequencing of 16S rRNA. Using the same sequencing approach, a reduction of *Ruminococcus* and *Blautia* species and an increase in the abundance of *Escherichia* were also found [9]. However, the more evident age-associated trait within the microbial population was the lower Firmicutes/Bacteroides ratio (F/B ratio) reported by Mariat et al. [49] via qPCR analysis and confirmed by Claesson et al. [9] by pyrosequencing of 16S rRNA. A schematic representation of the microbial changes associated with unhealthy microbiota occurring during aging and leading to host inflammation is depicted in Figure 1.

Most gut microbial changes observed during aging are attributable to diet composition. Both environmental and behavioral factors, including loss of sensation, tooth loss, chewing difficulties, changes in lifestyle, increased consumption of high sugar-fat foods and reduction in plant-based foods, and location of residence (community, long-term care, etc.), have been suggested to influence age-associated diet variations. Furthermore, reduced intestinal motility has been indicated to unfavorably affect gut fermentative processes in advanced age. Results from the ELDERMET project, aimed at investigating the association between diet, gut bacteria, and health status in a large cohort of elderly by pyrosequencing of 16S rRNA, showed that the setting of long-term care living represents a major factor affecting diet composition [9]. The authors identified a relationship between diet, microbiota, and health status. In particular, microbial population composition was mainly affected by the consumption of vegetables, fruits, and meat. Furthermore, in elderly people living in long-term care facilities, a higher proportion of Bacteroidetes was found compared with a higher Firmicutes population in community-dwelling persons within the same ethnogeographic region [9].

Taken as a whole, these results support a new hypothetical link between aging and microbiota alterations relying on a proinflammatory loop. In this context, the age-related decline in masticatory function together with a reduction of appetite and gastrointestinal motility induces dietary changes (reduction in fruits and vegetables) that is reflected in microbiota rearrangement (dysbiosis). This alteration, in turn, can activate a proinflammatory loop fueled by the immunosenescence of gut-associated lymphoid tissue releasing proinflammatory mediators which further favors microbiota rearrangements [50].

Regardless of diet, microbiota may also vary in older age in relation to several physiological and immunological statuses, such as antibiotic exposure [51, 52], decreased responsiveness of the immune system, and the existence of a chronic low-grade inflammatory status [53], as well as lifestyle and geographical location [54]. Indeed, bacterial 16S ribosomal RNA genes analyzed by next-generation sequencing in stool samples of Korean women aged 65+ with similar genetic background showed different gut microbial composition according to the location they are living, in island or inland areas [54]. Interestingly, the same approach on fecal samples of Italian elderly inpatients revealed an association between changes in microbial composition and polypharmacy, but not multimorbidity and frailty [55]. Notably, these changes were reported to impact mortality, rehospitalizations, and incident sepsis [55].

Besides the association with aging, dysbiosis has also been related to several undesirable conditions including obesity [56], inflammatory bowel disease [57], type 1 [58] and type 2 diabetes [59, 60], and nonalcoholic steatohepatitis [61] but has also recently been proposed to be involved in nonmetabolic syndromes such as age-related frailty [9], autism [62, 63], Alzheimer's disease [64], and depression [65].

## 3. The Importance of Dietary Supplementation on Microbiota

The analysis of microbial community composing human fecal samples of healthy individuals indicated that it is possible to distinguish the human gut microbiota into three main *enterotypes* based on the abundance of specific bacterial genera (i.e., *Bacteroides*, *Prevotella*, or members of the order Clostridiales) [66]. However, recent studies revisited this categorization and proposed the concept of bacterial communities being distributed as a continuum of abundance gradients between microbial genera [67].

Regardless of the exact microbial distribution, distortion of normal microbial balance has been implicated in several chronic conditions, including obesity and metabolic syndrome. Interestingly, antiaging strategies involving dietary manipulations addressing either variation in calorie intake or diet composition have been reported to affect the composition of gut microbiota. Changes in intestinal microbiota composition have been observed after weight loss following calorie restriction (CR), the only life-extending strategy available to date. In particular, an increase in the F/B ratio in obesity and a reduction of the same index with weight loss-producing CR-based interventions were found [68]. Obese people undergoing surgical (laparoscopic sleeve gastrectomy) or diet-based weight loss were also analyzed for changes in gut microbiota composition associated with weight loss interventions. Interestingly, in this case, differences in energy-reabsorbing potential were found to be associated with variation in F/B ratio [69]. A profile of weight gain-associated bacteria has been identified as related to the promotion of the expression of genes linked to carbohydrate and lipid metabolism thereby influencing dietary energy harvest [70]. Structural variations of gut microbiota have also been reported in animal models undergoing CR. For instance, a life-long low-fat diet significantly reshaped the overall structure of the intestinal microbiota in C57BL/6J mice. In particular, enrichment in phylotypes (genus *Lactobacillus*) positively correlated with longevity and a reduction in phylotypes negatively associated with lifespan was found in CR-treated mice [71].

Apart from strategies acting on calorie intake, diet composition (protein-rich versus fiber-rich dietary supplementation) represents a significant modulator of the microbial population of the gut [9, 72]. As such, diet is indicated as the main culprit responsible for metabolic diseases linked to gut dysbiosis. Even for short-term changes in consumption (4 days), animal-based and plant-based diets alter microbial community structure in a specific manner [72]. This change in food intake reflects the exchange between carbohydrate and protein fermentation existing between carnivore and herbivore mammals [72, 73].

Interestingly, Wu et al. [74], although reporting changes in microbiome composition within 24 h of high-fat/low-fiber or high-fiber/low-fat diet, showed that enterotype identity remained stable over 10 days of nutritional intervention. This suggests that food ingredients (e.g., dietary fibers) that are not digested by host enzymes but fermented by gut bacteria could modulate the gut microbiome composition in a relatively short period of time, independent of the effect of changes in transit time [74, 75]. This ability to resist disturbances and restore changes occurring in its composition (e.g., after short-term variations in dietary habits) is referred to as *resilience* [74, 75].

Beyond their primary role as dietary supplements ensuring the minimum nutritional requirements for maintenance and growth, some food components exert several beneficial effects on the host. This is achieved through the interaction with and modification of the gut microbiota. Among these, nutraceutical polyphenols, pre- and probiotics, vitamins, and polyunsaturated fatty acid (PUFA) supplementation have been recently investigated.

The administration of pre- and probiotics has been recommended as a dietary supplement to mitigate some of the age-related alterations in the intestinal microbiota associated with several gastrointestinal and respiratory diseases [76].

Probiotics defined as “live microorganisms which, when administered in adequate amounts, confer a health benefit on the host” [77] exert their beneficial effects on the host by improving gut barrier function, immunomodulation, and production of neurotransmitters as well as by modulating cellular components of the gut-brain axis [78]. On the other hand, prebiotics are “selectively fermented ingredient that allows specific changes, both in the composition and/or activity in the gastrointestinal microflora that confers benefits upon host well-being and health” [79].

The impact of probiotics, prebiotics, PUFAs, and phytochemicals, including flavonoids and phenolic compounds, on the gut microbiota is well characterized [78, 80–82].

Probiotics, particularly those containing *Bifidobacterium* and *Lactobacillus*, are among the most actively investigated microbiota-targeted interventions aimed at improving health status in advanced age [83, 84]. Results from a clinical trial showed that administration of *Lactobacillus rhamnosus* GG ATCC 53103 in healthy individuals, aged 65 to 80 years, was able to modulate the transcriptional response of the microbiota [85]. Oral supplementation of probiotics containing *Bifidobacterium brevis* B-3 and *Lactobacillus plantarum* HY7714, instead, has been shown to prevent skin photoaging induced by chronic ultraviolet irradiation in both mice and humans [86–88]. Similarly, oral administration of *Lactobacillus brevis* OW38 to aged mice ameliorated both age-associated colitis and memory impairments through the inhibition of lipopolysaccharide (LPS) production by the gut microbiota, p16 expression, and NF-*κ*B activation [89].

It is worth nothing that, when analyzing probiotic-mediated effects, host benefits are mediated through the promotion of microbiota homeostasis, rather than through changes in its composition [90].

Targeting gut microbiota has been indicated as a tool to modulate lean tissue mass. Bindels et al. showed that leukemic mouse model were cachectic mice with gut dysbiosis characterized by selective modulation of *Lactobacillus* spp. [91]. Following the administration of oral probiotic containing *Lactobacillus reuteri* and *L. gasseri*, an inverse association among serum levels of inflammatory cytokines [IL6 and monocyte chemoattractant protein-1 (MCP-1); the expression of protein associated with muscle atrophy, muscle RING-finger protein-1 (MuRF1); and atrogin-1] and muscle mass was found in these animals. Increased muscle mass and function (grip strength and swim time) have also been found in healthy young mice supplemented with *L. plantarum* [92]. The existence of a relationship between *Lactobacillus* species and skeletal muscle size found in this preclinical model needs to be confirmed in human studies.

The downside of probiotic usage including the potential risk of inducing gastrointestinal side effects, an unfavorable metabolic profile, excessive immune stimulation, and systemic infections in susceptible individuals, as well as horizontal gene transfer, needs also to be considered [93]. Therefore, a more comprehensive evaluation of the incidence and severity of adverse outcomes linked to probiotic consumption needs to be assessed.

Fermented nondigestible compounds, referred to as prebiotics, favor the proliferation of health-promoting bacteria [94] that may positively affect muscle health. Cani et al. [95] reported decreased levels of circulating LPS and inflammation and increased muscle mass in obese mice supplemented with prebiotics (i.e., fiber oligofructose) [95]. As a confirmation of the beneficial effect of prebiotic administration on gut microbiota, a shift in B/F ratio in addition to increased levels of *Lactobacillus* and *Bifidobacterium* spp. were found in follow-up analysis [96]. Further evidence supporting a link between prebiotic administration and effects on muscle mass is that association of proliferation of *Lactobacillus* and *Bifidobacterium* in leukemic mice with restoration of intestinal homeostasis (e.g., increase tight junction proteins) and reduced muscle wasting following administration of symbiotic inulin-type fructans and *Lactobacillus reuteri* [97]. These findings suggest that *Lactobacillus* and *Bifidobacterium* may influence gut-muscle communication and regulate muscle size. Interestingly, *Bifidobacterium* decrease with age [98] and are associated with lower circulating LPS levels [99]. Thus, an age-related decrease in gut *Bifidobacterium* content may underlie increases in circulating endotoxin that are shown to induce muscle atrophy [100]. While no conclusive data show increased muscle mass as an effect of *Bifidobacterium* supplementation especially in humans, there is evidence linking butyrate (associated with *Bifidobacterium* [101]) treatment as a protective strategy to counteract age-related muscle atrophy [102].

Indeed, 50+-year-old persons supplemented with galactooligosaccharides (GOS, 2 × 4 g/d for 3 weeks) in a randomized, double-blinded, placebo-controlled trial showed attenuation in age-associated *Bifidobacteria* reduction. In particular, an increase in the number of *Bifidobacteria*, together with higher *Lactobacilli* and butyrate levels, was obtained following GOS treatment. Moreover, SCFA concentration was increased whereas branched chain fatty acid concentrations were decreased by the same treatment. Thus, a more saccharolytic environment was achieved [103]. This and other studies based on GOS supplementation lead one to hypothesize that the administration of the GOS mixture in advanced age might positively affect the microbiota and age-associated markers of immune function [104].

The administration of symbiotic, comprising the probiotic *Bifidobacterium longum* and an inulin-based prebiotic component, has also been demonstrated to have an effect on the age-related changes in the intestinal microbiota. Indeed, an elevation in the number of *Bifidobacteria* as well as increasing members of the phyla Actinobacteria and Firmicutes together with a reduction of Proteobacteria was observed. Furthermore, treatment with this symbiotic caused an enhancement in butyrate production and a reduction in proinflammatory responses [105]. These findings might explain, at least in part, why probiotics have been successfully implemented as strategies to treat respiratory and gastrointestinal infections and enhance responses to vaccinations in older people [106].

Taken as a whole, these findings support the idea that pre- and/or probiotic supplementation may prevent age-related muscle loss by increasing the abundance of *Bifidobacterium* and butyrate producers in old individuals [85, 107].

Although the supply and conversion of nutrients are highly dependent on the composition of gut microbiota, bidirectional interactions between the microbiome, nutrient availability, and gastrointestinal function have also been proposed [108]. The metabolic activity of gut microbiota can modify the exposure, absorption, and potential health-promoting effects exerted by bioactive compounds, functional foods, or nutraceuticals.

Nutraceuticals are recognized among nutrients to beneficially modulate the growth, composition, and functions of the microbial host community in several animal models and recently also in humans [81, 109–112]. However, causality between bioactive compound assumptions and their benefits on host gut microbiota [113] is yet to be established and is challenging especially due to the complexity of endogenous and environmental factors affecting its equilibrium. Nevertheless, functional diets are proposed to prevent or attenuate metabolic diseases in view of their ability to elicit anti-inflammatory responses [114, 115].

As a whole, these findings support the hypothesis of a link between diet, microbiota, metabolism, and inflammation in several conditions and especially in advanced age [116]. Changes in microbiota have recently become the subject of intensive research because of their possible involvement in several conditions associated with inflammation, such as aging. However, a gap exists in the knowledge of how this could influence the variation of muscle mass and strength that accompanies aging. Here, we track some of the molecular pathways shared by age-related microbial alterations, metabolic changes, and sarcopenia in order to identify possible candidates and provide arguments in support of their exploitation in the management of muscle wasting.

## 4. Sarcopenia: A “Bacterial” Perspective

Muscle wasting is a key feature of several age-related conditions (e.g., sarcopenia, cachexia, and diabetes) leading to functional impairment and disability. Animal studies suggest a relationship between muscle wasting and alterations in the gut microbiome. Interestingly, muscle wasting induced in a mice model of acute leukemia was attenuated by oral supplementation with specific *Lactobacillus* species [91]. The authors suggested an influence of gut microbiota on muscle physiology through the regulation of amino acid availability.

Muscle protein metabolism is a multifactorial process resulting from the dynamic balance of protein synthesis and breakdown. Muscle protein synthesis is regulated by several anabolic stimuli (e.g., physical activity, food ingestion). Both amino acid (AA) composition of dietary proteins (e.g., prevalence of essential amino acids such as leucine) and their absorption kinetics (i.e., protein digestion speed and AA absorption) influence muscle protein synthesis [117]. Changes in the gut microbiota induced by clinically relevant interventions impact the bioavailability of dietary AAs [118]. Along the gastrointestinal tract, dietary and endogenous proteins are hydrolyzed into peptides and AAs by host- and bacteria-derived proteases and peptidases [119, 120]. The resulting peptides are subsequently released and support the growth and survival of bacteria in the gastrointestinal tract [121], but also regulate energy and protein homeostasis of the whole organism [122, 123]. AAs can also serve as precursors for the synthesis of SCFA by bacteria, thus suggesting an interplay between microbial activity and host AA and SCFA homeostasis [124]. The most abundant SCFA are propionate, butyrate, and acetate. The latter is mainly utilized by muscle cells to generate energy [125]. In addition to this, modulation of proinflammatory responses associated with microbial changes can be triggered by pathogens, various diseases, and malnutrition [126].

The presence of chronic, low-grade systemic inflammation, called “inflammaging,” also represents the substrate of aging and a highly significant risk factor for both morbidity and mortality in elderly people [127]. The inflammaging process is characterized by the persistent activation of innate immunity mediated by the NF-*κ*B transcription factor [128] and loss of CD4^+^ T cells.

Gut microbiota plays a crucial role in maintaining the balance of pro- and anti-inflammatory responses [129]. Aged gut microbiota may elicit an inflammatory response and display lower capability of counteracting adverse microbes or removing their metabolites [30]. The entrance of pathogens into the intestinal mucosa is also facilitated by the secretion of mucins by intestinal epithelial cells [130], which is triggered by a reduction in SCFA levels (especially acetate, n-butyrate, and n-propionate) in the intestines [30]. SCFA serves within the gut not only as an energy source for colonic epithelial cells but also as strong anti-inflammatory molecules regulating host metabolism and immunity [131]. In particular, butyrate modulates intestinal homeostasis through several actions, including the differentiation of CD4^+^ T cells into regulatory T cells, the induction of tumor growth factor- (TGF-) *β* secretion by epithelial cells, and the triggering of IL10 and retinoic acid production by dendritic cells and macrophages [131]. These actions allow for resolving local intestinal inflammation and avoiding its dissemination through leakage of bacteria and bacteria-derived inflammatory compounds into the blood [131].

Increased intestinal permeability to LPS is another element in support of a mechanistic link between microbial dysbiosis and systemic inflammation. Indeed, in young mice, high-fat feeding, which is known to compromise epithelial tight junctions and increase intestinal permeability [132], has been associated with decreased glucose tolerance and increased inflammation markers through LPS leakage from the intestine into the circulation [133].

In such a context, chronic inflammation may represent the *trait d'union* of microbial alterations and the development of muscle-wasting conditions in advanced age through a gut microbiota-muscle crosstalk. The molecular players involved in this process are not yet fully understood. Bäckhed et al. [134] showed that germ-free mice are protected from diet-induced obesity through increased fatty acid metabolism. This pathway involves AMP-activated protein kinase (AMPK), which monitors cellular energy status; increased muscular activity of carnitine:palmitoyltransferase-1 (CPT-1), which promotes the entry of long-chain fatty acylCoA into the mitochondria; and higher levels of the fasting-induced adipocyte factors linked to the peroxisome proliferator-activated receptor, gamma coactivator 1-alpha (PGC-1*α*), the regulator of mitochondrial content and oxidative metabolism. These increased activities counteract the impact of denervation and fasting on muscle atrophy.

The possible involvement of mitochondria in this crosstalk is not surprising if one considers that the maintenance of mitochondrial function is crucial to myocyte viability. Mitochondrial impairment and systemic inflammation play a central role in both cachexia and sarcopenia. Indeed, a role for proinflammatory cytokines [e.g., TNF-*α*, IL1*β*, IL6, TNF-like weak inducer of apoptosis (TWEAK)] in the induction of muscle catabolism has been previously reported [135].

Only one study has focused on the interface between chronic inflammation and mitochondrial clearance in skeletal muscle in the context of aging and physical frailty [136]. This investigation made use of IL10-null mice (IL-10tm/tm), a rodent model of chronic inflammation and frailty, and reported severe mitochondrial damage with disrupted organelle ultrastructure and abnormal autophagosomes in skeletal muscle [136]. Although these findings support the existence of a connection among mitochondrial dysfunction, cellular quality control failure, and inflammation, the signaling pathways responsible for such a link have yet to be fully elucidated. Circulating mtDNA is a prominent candidate for such a role, being an important damage-associated molecular pattern (DAMP) associated with inflammation and arising directly from mitochondrial damage [137].

**Figure 2:**
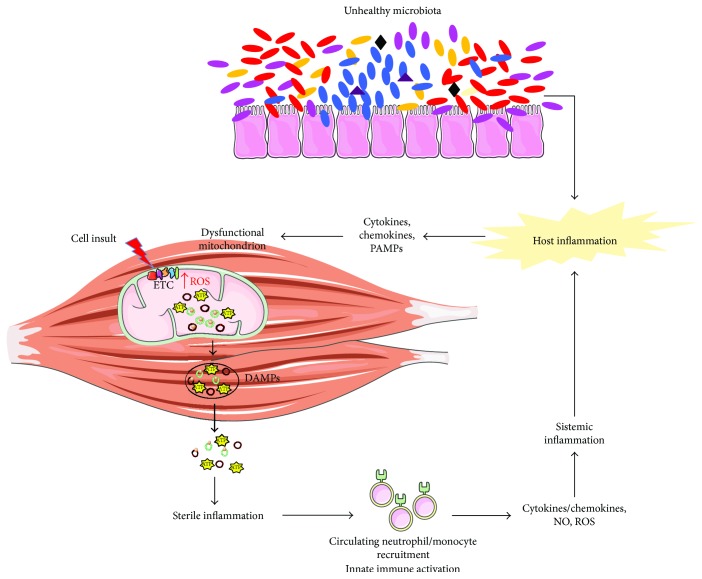
Proposed crosstalk between mitochondrial dysfunction and inflammation in muscle wasting. Imbalanced gut microbiota contributes to host inflammation and fuels the age-associated impairment of mitochondrial quality control in myocytes. This may lead to the release of mitochondrial damage-associated molecular patterns (DAMPs), such as mtDNA and ATP. The subsequent recruitment of local macrophages may maintain a persistent inflammatory milieu by alerting circulating immune cell and mounting a systemic response through the activation of mtDNA-induced inflammatory pathways. Cytokines, chemokines, nitric oxide (NO), and reactive oxygen species (ROS), released in the circulation by inflammatory cells, can induce further mitochondrial damage, thereby establishing a vicious circle and eventually contributing to muscle wasting. ETC: electron transport chain; mtDNA: mitochondrial DNA; TFAM: mitochondrial transcription factor A; PAMPs: pathogen-associated molecular patterns.

Recent findings by our group also support the idea that mitochondrial impairments in muscle occur in both sarcopenia and cachexia [138, 139]. Trigger candidates of inflammation in sarcopenia and cachexia could be represented by oxidized cell free-mtDNA or nucleoids extruded from damaged mitochondria (Figure 2). These DAMPs would activate the innate immune system and induce the subsequent production of inflammatory mediators. The release of the latter could sustain a vicious circle in myocytes through impaired quality control signaling, resulting in further mitochondrial impairment, increased reactive oxygen species generation, and the release of mitochondrial vesicles enriched with DAMPs. This series of events would fuel sterile inflammation, ultimately contributing to muscle wasting [140].

Due to its crucial role in host physiology and health status, age-related differences in the gut microbiota composition have been suggested to relate to the progression of diseases and frailty in old age. The first study correlating gut microbiota composition with frailty severity was conducted by van Tongeren et al. [141]. The authors demonstrated a significant reduction in the proportion of *Lactobacilli*, *Bacteroides/Prevotella*, and *Faecalibacterium prausnitzii* and an increase in the proportion of *Ruminococcus*, *Atopobium*, and Enterobacteriacae in older persons with high frailty scores [141].

The finding of dysbiotic shifts of gut microbiota towards a greater abundance of butyrate-producing bacteria such as *Faecalibacterium prausnitzii* in higher functioning persons suggests a positive role for these microbes in muscle function. Indeed, butyrate, by enhancing intestinal barrier function through the reinforcement of tight junction assembly [142], should prevent endotoxin translocation and reduce circulating inflammation [143].

Evidence from metagenomic analysis in a large sample of older adults (*n* = 178), the ELDERMET study, clearly linked butyrate-generating bacteria with functional capacity by showing that community-dwelling elderly have more butyrate-producing microbes than those in long-stay residence [9]. This finding, together with a greater abundance of Enterobacteriaceae and *Escherichia/Shigella* and reduced gut microbial diversity among institutionalized elderly, highlights the need of nutritional strategies aimed at preventing the loss of “healthy” microbes (e.g., butyrate-producing bacteria) for those individuals entering long-term care facilities. Notably, prebiotic supplementation (inulin plus fructooligosaccharides) has been shown to increase muscle strength and endurance in frail older adults [144], thereby highlighting the potential of prebiotic supplementation as a treatment for age-associated deficits in muscle function.

Such findings, although indicating gut microbial changes among the factors affecting muscle mass and quality during aging, are not yet conclusive. Further research aimed at deciphering the pathways involved in microbiota-immune system crosstalk and its implication in muscle aging is warranted.

## 5. Catching the Microbiota Complexity: Opportunities from Next-Generation Sequencing Approaches

The advent of sequencing technologies has revolutionized the analysis of complex microbiomes and their functions and has allowed for upgrading fundamental theories of evolution [145].

The next-generation sequencing (NGS) revolution has enabled the genomic and functional characterization of novel microbial species, especially pathogens, revealing the diverse composition of microbial communities in several environments and the association of microbial groups with specific activities [146]. Technological advances in sequencing platforms have ensured increasingly long-read lengths that have helped cut down the cost of sequencing, one of the major limitations of the technology [147]. This has led to a dramatic increase in the amount of sequencing data generated. Such a burst in big data production and the parallel exponential increase in computational power have introduced new challenges and bottlenecks related to handling the complexity of the information generated and storing it [148, 149], especially in medicine [150].

Metagenomic studies, among other methods, have taken advantage of increasing computational power to address more complex questions compared with traditional genomic approaches.

Since its inception in 1998 [151], metagenomics has allowed for culture-independent analysis of several complex microbial populations, thus capturing the variability of microbial ecosystems that could not be identified under standard laboratory conditions [152]. This approach has revealed structural diversity, functionality, microbial interactions with the environment, other microbes and the host, and evolutionary processes [13, 153–155].

Targeted metagenomics, known as metagenetic [156], is based on marker gene amplification and the sequencing of 16S ribosomal RNA gene (16S libraries), the domain of which is restricted to *Bacteria* and *Archaea* [157]. Whole-metagenome shotgun analyses, instead, are accomplished by unrestricted sequencing of the collective microbial genomes present in the sample (shotgun libraries). While 16S sequencing approach aims at reconstructing the taxonomic content of the microbial population, the shotgun approach can address the question of how the collective microbial genomes interact in the sample. This allows for a functional microbial characterization by retrieving the complete sequences of protein-coding genes in the sequenced genomes [158]. The choice between these two methods depends on the nature of the study. The 16S approach is generally used with large sample sizes and in longitudinal studies, while the shotgun approach is preferred when a functional characterization within the samples is required [159].

Although being more expensive, the shotgun approach generates more informative libraries when performed with appropriate sequencing depth [154, 160]. Li et al. [161] published a nonredundant reference catalogue of 9,879,896 genes by combining 249 newly sequenced samples in the Metagenomics of the Human Intestinal Tract (MetaHit) project with 1018 previously sequenced samples (data available at http://meta.genomics.cn). Likewise, a shotgun approach was recently used by Xie et al. [162] to construct a comprehensive gut microbial reference gene catalogue from a metagenomic analysis of fecal samples of 250 adult twins from the UK. In this study, the authors demonstrated the heritability of many microbial taxa and functional modules in the gut microbiome, including disease-associated ones. However, the application of shotgun metagenomics to overcome the limited taxonomic resolution and functional inference of metagenetic approaches and to reveal the functional association of gut microbiota in disease conditions is still limited [163].

Especially in relation to human health, the study of 16S is of critical importance, since several disease conditions have been associated with decreased microbiome diversity or with the abundance of specific microbial species. The binning process, which is defined as the assignment of sequences to the corresponding taxonomic group, referred to as operational taxonomic unit (OTU), is pivotal in defining the diversity of the sample and its taxonomic composition. In addition, it facilitates genome assembly and the evaluation of gene association with different taxonomic groups and derived metatranscriptomic or metabolomic analyses [164, 165]. The binning process, the accuracy of which depends mainly on the clustering algorithm and on the preprocessing of the reads [166], is usually carried out with taxonomy-dependent and independent methods: the first performs a standard homology inference against a reference database to classify DNA fragments [167–171], while the second is a reference-free method which applies clustering techniques on features extracted from the sequences [172–176].

Though, when assessing gut microbiota composition by 16S analysis, many sources of bias have been recognized. These include adequacy of the experimental design and data analysis. In particular, the choice of the extraction kit [177], primer selection and hence the regions to be amplified [178], library preparation methodology [179], sequencing errors [180], and sequencing throughput as well as the choice of pipelines and reference databases for data analysis [181] strongly impact the results.

For all these reasons, the need for a standardized method is required in order to compare datasets generated by different platforms, especially for clinical and diagnostic purposes.

Despite these criticalities, large-scale projects [The NIH Human Microbiome Project, the Metagenomes of the Human Intestinal Tract (MetaHIT), and the ELDERMET project] succeeded in paving the way to a comprehensive determination of the microbial composition of the gut microbiota and its relationship with health and diseases [9, 16, 161, 182–184]. These findings are further supported by advanced computational tools and dedicated pipelines for the analysis of microbial community data [181, 185, 186] including mothur [187], w.A.T.E.R.S [188], the RDP classifier [189], mOTU [190], and QIIME [191], defined as the “gold standard” for 16S metagenomic datasets [192].

Several software programs have been developed to infer metabolic capacity and functionality from 16S libraries. PICRUSt (Phylogenetic Investigation of Communities by Reconstruction of Unobserved States) is the first and most used software that associates representative sequences from OTUs to nodes of a reference phylogenetic tree [193]. In addition, it can predict gene content even in sequenced genomes not available by using ancestral state reconstruction algorithms. Other examples are Tax4Fun [194], which relies on the KEGG database, the SILVA SSU Ref NR database, and Piphillin [195], which has implemented an inference tool that works with any current genome database and has improved correlation and accuracy for clinical samples compared with PICRUSt and Tax4Fun [166].

Metagenomics is one of the most powerful tools available to unravel the complexity of gut microbiota. The integration of metagenomic data and other “omic” techniques (e.g., proteomics, metabolomics), within a multidimensional approach, will be crucial to define the determinants of several clinical conditions and thus identify complementary biomarkers [196, 197] and new therapeutic targets [198] based on nutritional and transplantation interventions.

## 6. Conclusion and Future Perspectives

The identification of specific biomarkers that may aid in the development of noninvasive tools for the assessment and monitoring of the relationship between inflammation and muscle wasting conditions has been sought for a long time. Current research efforts on specific “danger molecules” that stimulate sterile inflammation and link this process with muscular mitochondrial dysfunction could enhance our understanding of muscle wasting pathophysiology. Results from several studies indicate the relevant contribution of microbial changes and activity in the gut to the repertoire of inflammatory molecules involved in the *milieu* characterizing muscle aging. This represents an important matter to be addressed by future investigations to unravel the signaling pathways that may serve as targets for interventions.
